# Rlip76: An Unexplored Player in Neurodegeneration and Alzheimer’s Disease?

**DOI:** 10.3390/ijms23116098

**Published:** 2022-05-29

**Authors:** Ashly Hindle, Sharda P. Singh, Jangampalli Adi Pradeepkiran, Chhanda Bose, Murali Vijayan, Sudhir Kshirsagar, Neha A. Sawant, P. Hemachandra Reddy

**Affiliations:** 1 Department of Internal Medicine, Texas Tech University Health Sciences Center, Lubbock, TX 79430, USA; ashly.hindle@ttuhsc.edu (A.H.); sharda.singh@ttuhsc.edu (S.P.S.); pradeep.jangampalli@ttuhsc.edu (J.A.P.); chhanda.bose@ttuhsc.edu (C.B.); murali.vijayan@ttuhsc.edu (M.V.); sudhir.kshirsagar@ttuhsc.edu (S.K.); neha.sawant@ttuhsc.edu (N.A.S.); 2 Neuroscience & Pharmacology, Texas Tech University Health Sciences Center, Lubbock, TX 79430, USA; 3 Neurology, Departments of School of Medicine, Texas Tech University Health Sciences Center, Lubbock, TX 79430, USA; 4 Public Health Department of Graduate School of Biomedical Sciences, Texas Tech University Health Sciences Center, Lubbock, TX 79430, USA; 5 Department of Speech, Language and Hearing Sciences, School Health Professions, Texas Tech University Health Sciences Center, Lubbock, TX 79430, USA

**Keywords:** RALBP1, Rlip, neurodegeneration, Alzheimer’s disease, oxidative stress, mitochondrial dysfunction

## Abstract

Alzheimer’s disease (AD) is a progressive neurodegenerative disorder and is the most common cause of dementia in older people. AD is associated with the loss of synapses, oxidative stress, mitochondrial structural and functional abnormalities, microRNA deregulation, inflammatory responses, neuronal loss, accumulation of amyloid-beta (Aβ) and phosphorylated tau (p-tau). AD occurs in two forms: early onset, familial AD and late-onset, sporadic AD. Causal factors are still unknown for a vast majority of AD patients. Genetic polymorphisms are proposed to contribute to late-onset AD via age-dependent increases in oxidative stress and mitochondrial abnormalities. Recent research from our lab revealed that reduced levels of Rlip76 induce oxidative stress, mitochondrial dysfunction and synaptic damage, leading to molecular and behavioral phenotypes resembling late-onset AD. Rlip76 is a multifunctional 76 kDa protein encoded by the *RALBP1* gene, located on chromosome 18. Rlip is a stress-protective ATPase of the mercapturic acid pathway that couples clathrin-dependent endocytosis with the efflux of glutathione–electrophile conjugates. Rlip is evolutionarily highly conserved across species and is ubiquitously expressed in all tissues, including AD-affected brain regions, the cerebral cortex and hippocampus, where highly active neuronal metabolisms render the cells highly susceptible to intracellular oxidative damage. In the current article, we summarize molecular and cellular features of Rlip and how depleted Rlip may exacerbate oxidative stress, mitochondrial dysfunction and synaptic damage in AD. We also discuss the possible role of Rlip in aspects of learning and memory via axonal growth, dendritic remodeling, and receptor regulation. We conclude with a discussion of the potential for the contribution of genetic polymorphisms in Rlip to AD progression and the potential for Rlip-based therapies.

## 1. Introduction

Currently there are no good therapeutic options for curing or slowing the progression of a variety of cognitive disorders, and this is especially true for neurodegenerative disorders such as Alzheimer’s disease (AD), vascular dementia (VD), Huntington’s disease (HD), Parkinson’s disease (PD), and treatment-refractory epilepsy, which together affect over 8 million Americans. Drugs may aid in the management of symptoms, for example levodopa for PD and tetrabenazine for HD, but the underlying neurodegenerative trajectories remain unaltered. Improved molecular understanding and new neuroprotective therapies to delay or prevent these progressive diseases are urgently needed.

Rlip76 (Rlip) is a multifunctional 76 kDa protein encoded by the RalA Binding Protein 1 (*RALBP1*) gene on chromosome 18. Rlip is a stress-protective ATPase of the mercapturic acid pathway that couples clathrin-dependent endocytosis (CDE) with the efflux of glutathione-electrophile conjugates (GS-Es) [1,2,3]. Rlip was independently discovered by the labs of Drs. Yogesh Awasthi, Larry Feig, Jacques Camonis, and Robert Weinberg. Rlip was first described by the Awasthi lab in 1992 as an ATP-dependent transporter of 2,4-dinitrophenyl-S-glutathione (DNP-SG, glutathione conjugate of 1-chloro-2,4-dinitrobenzene) and leukotrienes in erythrocytes, muscle, and liver. These are tissues in which the high levels of iron lead to the production of reactive oxygen species (ROS), which can cause intracellular and extracellular damage [4,5,6,7]. Then, in 1995, three research groups cloned a novel Ral pathway protein using yeast two-hybrid assays [8,9] and radiolabeled Ral-based screening of an expression library [10]. Within a few months of each other the Feig lab described their discovery of RALBP1 (Ral-Binding Protein 1), the Camonis lab described RLIP76 (Ral Interacting Protein, 76 kDa), and the Weinberg lab described RIP1 (Ral Interacting Protein 1) [8,9,10]. RALBP1/RLIP76/RIP1 was found to preferentially bind Ral-GTP over Ral-GDP, and early characterization efforts also found that it possessed GAP (GTPase Activating Protein) activity toward Rac1 and Cdc42 [8,9]. The Awasthi lab later confirmed that DNP-SG ATPase was in fact the same protein as RALBP1/RLIP76/RIP1 (referred to hereafter as Rlip) [11]. Since that time, Rlip has been shown to directly mediate or participate in pathways which mediate oxidative stress response, receptor internalization, mitochondrial fission, and plasma membrane outgrowth, among other functions. Rlip is highly conserved across species [12] and is ubiquitously expressed in all tissues, including the brain [13]. Neurons have several characteristics which would make them relatively susceptible to disruptions of the functions in which Rlip participates. These include: (1) a high metabolism and a high energy requirement to maintain membrane potential for action potentials, (2) susceptibility to oxidative damage, (3) a high dependence on endocytosis and exocytosis for neurotransmission, and (4) dependence on cytoskeletal-mediated dynamic membrane reorganization for neurite outgrowth. However, despite the fact that Rlip participates in several cellular functions which are particularly important for neuronal activity, the role of Rlip in neurological disorders has been relatively unexplored.

Recently, we reported that mice with a heterozygous loss of Rlip (Rlip^+/−^) showed cognitive impairments, increased oxidative stress, and mitochondrial abnormalities, features similar to those seen in late-onset AD [14]. Molecular markers of synaptic function, as well as mitochondrial fission, fusion, and biogenesis, were also affected in brain tissues obtained from these mice [14]. Transmission electron microscopy studies of cortical and hippocampal tissues revealed abnormalities in both the size and number of mitochondria. Gene network analysis indicated dysregulated expression of stress-activated genes, mitochondrial function genes, and CREB signaling genes in the Rlip^+/−^ mouse brain. Taken together, our results suggested that Rlip deficiency was associated with increases in oxidative stress and mitochondrial dysfunction, factors that may contribute to the progression of oxidative stress-related neurodegenerative diseases such as AD. Significantly, these findings also show that a halving of Rlip gene dose by heterozygous Rlip knockout (KO) is sufficient to induce behavioral alterations and markers of oxidative stress and mitochondrial dysfunction in the brain. Thus, complete Rlip KO was not required to induce the aberrant phenotype, suggesting that a partial reduction of Rlip expression may be sufficient to induce a cognitive decline in AD.

Building on these findings, the goal of this mini-review is to detail the structure, function and cellular pathways of Rlip, discuss the importance of those pathways to neuronal function, and detail how changes in those functions may link to brain function in healthy and disease states, with primary focuses on AD, oxidative stress, mitochondrial abnormalities, and synaptic damage. We end by discussing the possibility that genetic polymorphisms in Rlip may have a link to AD.

## 2. Rlip Structure and Function

**Figure 1:**
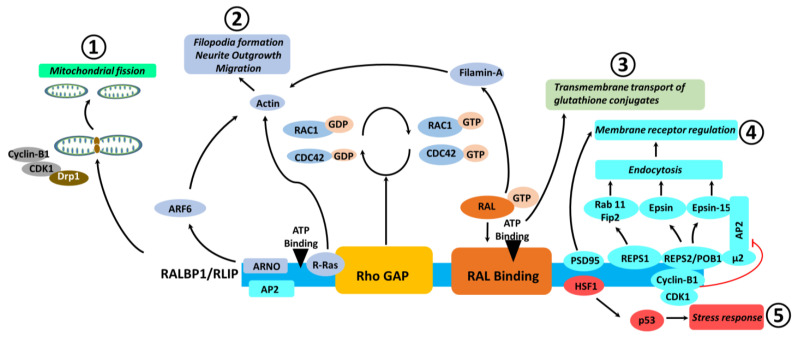
Structure, interactions, and functions of Rlip. Rlip is a 76 kDa multi-domain protein which carries out or regulates a variety of functions. The major resulting endpoints of Rlip activity are enumerated and illustrated as separate pathways. These include (**1**) mitochondrial fission, (**2**) membrane remodeling activities, (**3**) efflux of glutathione conjugates, (**4**) receptor regulation via endocytosis, and (**5**) stress response.

Structurally, Rlip has several distinct and overlapping interaction regions, which have been nicely summarized by Cornish et al. (2021) in a recent review article [15]. Several of these interactions are illustrated in Figure 1. Starting from the N terminus, Rlip has a region which interacts with the AP2 clathrin adapter complex and with ARNO, proteins which facilitate endocytosis and cell spreading/migration, respectively [15,16]. Moving in the N→C direction, next comes a short region that binds R-Ras, an interaction which contributes to ARNO activation. Encompassing both the AP2/ARNO interaction region and the R-Ras interaction residues is a region shown to bind Epsin, an interaction which is also thought to regulate cell migration, along with the shutdown of clathrin-dependent endocytosis during mitosis [17,18]. We then encounter the RhoGAP domain, which interacts with the Rho family GTPases Rac1 and Cdc42, proteins which regulate cytoskeletal dynamics, migration, cell cycling, and clathrin-dependent endocytosis, among a wide array of other functions [16,19,20,21]. This is followed by the Ral binding domain through which Rlip carries out effector functions downstream of both RalA and RalB [15]. Rlip-RalA and Rlip-RalB interactions have been reported to contribute to several functions including the regulation of mitochondrial fission at mitosis [22], clathrin-dependent endocytosis of receptors [23], p27 localization [24], cytoskeletal remodeling [25], and invadopodia formation [26].

A commonality among many of these functions is that they require the application of mechanical forces on a membrane. In the case of CDE, the actin cytoskeleton is important for providing the force required to constrict and separate vesicles from the plasma membrane [27]. In the case of mitochondrial fission, dynamin-related protein 1 (Drp1) oligomers surround and mechanically constrict the mitochondria [28], while plasma membrane outgrowths such invadopodia and neurites require forces transmitted through actin bundles [29,30]. For the sake of comprehensiveness, it is also worth noting that Rlip is frequently bound to tubulin [31], and a *RALBP1* splice variant called cytocentrin functions in spindle separation during mitosis, a process in which microtubules play a key role [32]. In keeping with Rlip’s other functions, spindle separation is also a process requiring mechanical forces generated against cytoskeletal scaffolding [33]. The C-terminal third of Rlip consists of a predicted coiled coil region which interacts with POB1, REPS1, HSF1, and PSD95 [15]. Rlip contains two ATP binding sites which resemble the Walker motif (^69^GKKKGK^74^ and ^418^GGIKDLSK^425^) [34]. Mutation of lysines K74 and K425 to methionines was found to abrogate the ATP hydrolysis activity of Rlip and diminish xenobiotic transport [34], and the formation of invadopodia was abrogated in cells expressing Rlip with the K425M mutation, relative to wild-type [26].

Overall, Rlip has several structural and functional domains related to oxidative stress and mitochondrial function that maintains cellular homeostasis, and a reduction of Rlip induces oxidative stress and mitochondrial dysfunction in neurodegenerative diseases such as AD. Our initial studies revealed that a partial reduction of Rlip induced oxidative stress and mitochondrial damage in AD [14]. However, further research is still needed in order to determine the precise domain structural involvement in oxidative stress/mitochondrial function in AD.

## 3. Stress Responsiveness

Rlip shows stress-responsive changes in activity [31] and has been found to bind to the master stress-response transcription factors p53 and HSF1 (heat shock factor 1) [31,35]. p53 is generally characterized as the ‘guardian of the genome’ which regulates responses to genotoxic stress [36]. HSF1 regulates the response to proteotoxic stressors including temperature and oxidative damage; however, there is considerable overlap in the conditions which activate p53 and HSF1 [36]. This overlap is to be expected, in part because reactive oxygen species cause DNA damage, and in part because HSF1 can translocate p53 into the nucleus [37]. Hu et al. (2003) found that when cells are in an unstressed state cytosolic Rlip associates with HSF1, HSP90, and tubulin in heterocomplexes [31], and Singhal et al. (2008) found that plasma membrane Rlip forms a ternary complex with HSF1 and POB1 [38]. In the unstressed state, HSF1 and Rlip are mutually inhibitory, with HSF1 inhibiting the efflux function of Rlip, and Rlip sequestering HSF1 in the cytosol. Under stress, Rlip and HSF1 dissociate [31,38], freeing Rlip to catalyze the ATP-dependent efflux of glutathione conjugates and xenobiotics and freeing HSF1 to translocate to the nucleus to activate stress response pathways [31,38]. Interestingly, Singhal et al. reported that stress also induces Rlip translocation to the nucleus, suggesting that Rlip may also have a role in transcriptional regulation [38]. This is supported by recent findings that Rlip depletion by phosphorothioated DNA antisense results in broad methylomic and transcriptomic changes in the livers of p53 KO mice, relative to controls treated with scrambled phosphorothioate [39]. It has also been shown that p53 binds to membrane Rlip, inhibiting the transport of glutathione-conjugated 4-hydroxynonenal (4-HNE) and doxorubicin [35]. It is well-established that during normal unstressed conditions p53 degradation is rapidly induced by MDM2, and that following an exposure to genotoxic stress this degradation is halted, causing a rapid rise in p53 [40]. The authors interpreted this inhibition of Rlip by p53 as a possible mechanism by which p53 induces apoptosis in cells challenged by overwhelming genotoxic stress, rather than a means of basal state Rlip inhibition, as observed with HSF1–Rlip interactions. Finally, neuroinflammation is a key component of the current models of AD pathogenesis [41]. In keeping with Rlip’s role as a stress responsive protein, it has been reported that TNFα induces Rlip expression in blood brain barrier endothelial cells during inflammation [42]. Further study is needed to confirm whether this also occurs in neurons; however, it is clear that TNFα is expressed in neurons during neuroinflammation [43]. Since Rlip functions to increase stress tolerance it is conceivable that a reduction in Rlip, or a failure to appropriately upregulate Rlip expression, could indirectly exacerbate the detrimental effects of neuroinflammation. In a clinical context, therapeutic increases in Rlip may confer neuronal stress tolerance, helping to slow neurodegenerative progression. The major side effect of concern from increased stress tolerance would be the increased resistance of premalignant cells to elevated metabolic stresses. This may facilitate proliferation and increase the risk of cancer. For this reason, it may be wise to concentrate potential therapeutic development efforts on strategies which target the CNS, rather than on systemic therapies.

Overall, these interactions indicate that Rlip, p53, HSF1, and TNFα work together to coordinate the cell response to several types of stress, including oxidative stress. Oxidative stress from ROS released by dysfunctional mitochondria is a major component of current models of AD pathogenesis. Thus, it is reasonable to hypothesize that impairments in the activity of Rlip may cause sub-optimal responses to oxidative or genotoxic stresses, neuroinflammation, and the exacerbation of neuronal damage.

## 4. Energy Production: Rlip in Mitochondrial Biogenesis

Neurons are highly metabolic. The firing of action potentials and the maintenance of membrane potentials require an abundance of energy, with Na+/K+-ATPase accounting for up to two-thirds of a neuron’s energy demand [44]. Glucose is the preferred source of energy, and the brain has been shown to consume 20% of the body’s energy, primarily as glucose, despite comprising only 2% of the body’s mass [45]. Thus, impaired energy production is highly problematic for neurons. Dysfunctions of the mitochondria, which produce the majority of cellular ATP through oxidative phosphorylation, have been shown to contribute to the aberrant neurodegeneration in Alzheimer’s disease, and mitochondrial-targeted drugs are promising candidates among the therapies under development [46,47,48].

**Figure 2:**
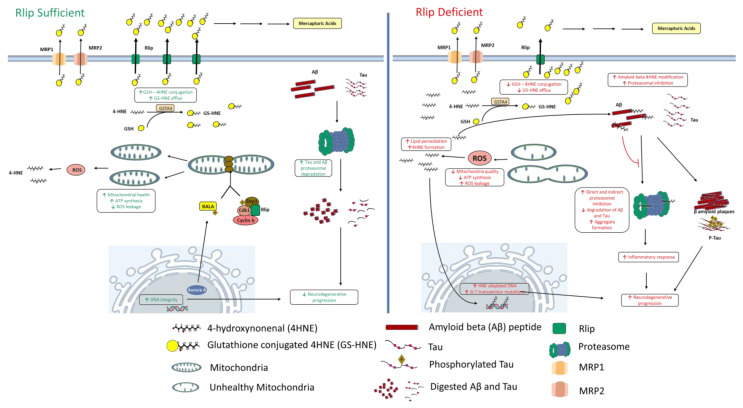
Model of Rlip in mitochondrial health, oxidative stress and DNA damage. The left panel depicts cellular conditions when Rlip is abundant. Under this scenario, mitochondria are healthy and producing minimal ROS, 4-HNE is cleared from the cell, Aβ and Tau are degraded, and the DNA is not subjected to insult. The right panel depicts cellular conditions of Rlip deficiency. Unhealthy mitochondria emit greater ROS, leading to increased 4-HNE production. This 4-HNE is inefficiently cleared from the cell, causing accumulation. Accumulated 4-HNE can cause adducts on DNA, proteasomes, and Aβ. 4-HNE adducts on both proteasomes and on Aβ can inhibit proteasomal function, inhibiting Aβ and Tau degradation and causing inflammation [50,51,52,53,54]. Aβ and p-Tau aggregation, along with neuroinflammation, can exacerbate neurodegeneration and progression to AD.

Mitochondrial fission at mitosis has been reported to involve Rlip [22,49]. An illustration of this can be seen in Figure 2. Phosphorylation of RalA by Aurora A kinase localizes RalA and Rlip to the mitochondria, where Rlip serves as a scaffold to recruit cyclin B/Cdk1 to the mitochondria, where they in turn phosphorylate and recruit the mitochondrial fission protein Drp1 [22]. It has also been found that RalA and RalB promote the clearance of dysfunctional mitochondria from the cell by mitophagy. Following the depolarization of a mitochondria, RalA and RalB relocalize to the mitochondria where they activate TBK1 which in turn promotes mitophagy [50]. Interestingly, this relocation of RalA and RalB to the mitochondria appears to be linked to CDE [50]. Because Rlip is both an RAL effector and required for CDE, it likely also plays a role in this process, although this has not been experimentally verified.

Our recent findings show that heterozygous Rlip KO (Rlip^+/−^) mice have significant increases in the dentate gyrus and hippocampus expression of Drp1 and Fis1, genes involved with mitochondrial fission, and decreased expression of the mitofusin MFN1 [14]. Electron microscopy images of hippocampal and cortical cells in Rlip^+/−^ mice showed a larger number of smaller mitochondrial relative to controls with wild-type Rlip, indicating mitochondrial fragmentation [14]. Consistently, the activity of glutathione peroxidase, a mitochondrial antioxidant enzyme, decreased in the Rlip^+/−^ mice, which also indicates a loss of mitochondrial function [14]. Together, these results suggest that the mitochondria of Rlip^+/−^ mice may be dysfunctional, with impaired energy production and elevated leakage of ROS into the surrounding cytosol. Supporting a possible energy deficiency, homozygous Rlip knockout mice, while viable, tend to remain small and thin. Sperm motility is also highly dependent on mitochondrial ATP production [55], and although we have not formally quantified it, we have clearly observed that homozygous Rlip knockout mice have reduced fertility relative to wild-type littermates. In summary, evidence from the literature suggests that Rlip plays a role in the maintenance of healthy mitochondria, which are key to producing the energy which is critically needed by neurons. We posit that Rlip deficiency may therefore contribute to reduced mitochondrial ATP production, perhaps affecting neuronal vitality and contributing to neurodegenerative diseases.

## 5. 4-HNE, Oxidative Stress, and Neurodegeneration

In AD patients, amyloid β (Aβ) peptides induce oxidative stress by coordinating reactive metal ions which can catalyze ROS formation and lead to a cascading series of detrimental events [56]. Following its formation, ROS can initiate the peroxidation of polyunsaturated fatty acids, leading to production of 4-HNE. 4-HNE is a genotoxic and proteotoxic peroxidation product of omega-6 polyunsaturated fatty acids (PUFAs) such as arachidonic acid and linoleic acid [11]. Indeed, the brains of Alzheimer’s disease patients have high levels of cerebrospinal fluid 4-HNE and increased 4-HNE adducts on neurofilaments and amyloid β plaques [57]. 4-HNE-modified amyloid β peptides have been found to inhibit proteasomal function, leading to neuroinflammation which exacerbates neurodegeneration [51,53]. In Parkinson’s patients, direct alkylation of the 26S proteasomal subunit by 4-HNE has been associated with loss of proteasomal function, neurodegeneration, and further elevated oxidative stress [52]. To our knowledge, this proteasomal alkylation effect has not been directly demonstrated in AD models, but it has also been shown to occur in rat livers [58], and there is every reason to suspect that proteasomes found in AD brains would also be subject to the same alkylation. It is also known that tau is cleared by proteasomal degradation [54] and that 4-HNE inhibits the dephosphorylation of tau protein [59]. These support a model whereby elevated 4-HNE would also increase the levels of total tau, p-tau, and consequently, increase the formation of neurofibrillary tangles. These effects are illustrated in the Rlip-deficiency panel of Figure 2.

Beyond the oxidative stress generated as a byproduct of neuronal metabolism and the aberrant ROS production catalyzed by amyloid β peptide, there is also evidence that unhealthy mitochondria emit higher levels of ROS, increasing oxidative stress and lipid peroxidation, and causing damage to mitochondrial and nuclear DNA [60,61,62]. It is thought that this further exacerbates the mitochondrial dysfunction in a vicious positive feedback cycle; however attempts to experimentally confirm such a model have yielded mixed results [63]. Several lines of evidence support a key role for Rlip in the detoxification of 4-HNE and response to oxidative stress [14,64,65], and thus the downregulation or degradation of Rlip may increase the damage caused by the elevated ROS emission resulting from amyloid β and mitochondrial dysfunction. There is also evidence that mitochondrial ROS can trigger the production of amyloid β, which can in turn further damage mitochondria [66,67], providing an additional layer to positive feedback models.

## 6. Rlip in the Prevention of Oxidative Stress

As a byproduct of their high metabolic demands neurons must detoxify or remove a large amount of reactive oxygen species [68]. Rlip was originally characterized as a DNP-SG ATPase purified from erythrocytes, which are highly susceptible to oxidative damage due to the abundant heme iron of hemoglobin. To protect against oxidative stress, erythrocytes contain high levels of glutathione (GSH) [69]. GSH is the dominant cellular antioxidant used in detoxifying reactive oxygen species and toxic electrophiles derived from lipid peroxidation [70,71]. It was later shown that Rlip is required for the low-affinity high-capacity efflux of the glutathione conjugate of 4-HNE, a genotoxic and proteotoxic peroxidation product of omega-6 polyunsaturated fatty acids (PUFAs) such as arachidonic acid and linoleic acid [11]. 4-HNE can reach levels as high as 1 µM in normal human plasma [72]; however, concentrations as high as 100 µM have been observed near the site of lipid peroxidation [57]. Rlip’s role can thus be conceptualized as a pressure relief valve which mitigates the destructive potential of high levels of oxidative stress. Its low affinity suggests that when low levels of 4-HNE are present, detoxification will be controlled by higher affinity transporters such as MRP1 and MRP2 [73], while at high ROS burdens Rlip-mediated efflux can quickly remove a large quantity of glutathione conjugates, serving not only to clear toxins from the cells but also to prevent the glutathione transferase-mediated conjugation reaction from backing up due end product accumulation [74]. As a consequence of this, diminished Rlip expression or activity may elevate 4-HNE even when the MRP1 and MRP2 transporter systems are intact. In Figure 2, this is depicted as an increased accumulation of free 4-HNE on the right (Rlip-deficiency) panel.

In the introduction, we described our findings that the cortical and hippocampal mitochondria are abnormal in Rlip^+/−^ mice, which likely indicates suboptimal energy production by oxidative phosphorylation and increased ROS leakage from the mitochondria of these neurons. Consistent with elevated ROS, in the brains of Rlip^+/−^ mice we found increased expression of NRF2 [14], a master transcription factor which is activated by oxidative stress and which subsequently induces the cell’s antioxidant system. This also supports an increase in oxidative stress in the brains of mice with reduced Rlip. Taken together, it is likely that Rlip plays a key role in the management of oxidative stress in neurons. Thus, loss of Rlip may elevate oxidative stress via both the increased release of ROS and decreased detoxification of oxidative fatty acid metabolites like 4-HNE. The detrimental effects of 4-HNE in neurons, combined with the established role of Rlip in the cellular efflux of 4-HNE, are alone sufficient to argue the need for further study of Rlip in the context of neurodegenerative disorders.

## 7. Rlip in the Prevention of DNA Damage

Damage to mitochondrial and nuclear DNA is a recognized problem in the progression of AD and other neurodegenerative disorders. Oxidative DNA damage and mitochondrial dysfunction are thought to contribute to the neurodegeneration of AD [75,76], vascular dementia [77], HD [78], PD [79] and epilepsy [80]. Because oxidative stress contributes to DNA damage, it is possible that reduced Rlip expression or loss of Rlip functionality increases DNA damage in neurons (Figure 2). Rlip expression is induced by oxidative stress, and Rlip serves as one of the key efflux pumps for removing 4-HNE [64]. Following conjugation of 4-HNE to glutathione (GSH) by GSTA4, the resulting GS-HNE molecule is transported from the cell by Rlip in an ATP-dependent process. This is an important process because 4-HNE is highly reactive and mutagenic, resulting in DNA adducts, particularly on guanines [81], and Rlip depletion has been found to increase the accumulation of 4-HNE and its adducts. 4-HNE adducts can alter DNA base pairing, resulting in stabilized G^HNE^-A mispairs, which can give rise to G-C→T-A transversion mutations [82]. 4-HNE has also been shown to result in a low level of interstrand crosslinks, which are the most destructive type of DNA adduct [82]. Finally, 4-HNE has also been found to decrease the activity of the DNA repair enzyme 8-oxoguanine glycosylase-1 (OGG-1), possibly due to covalent alkylation of the protein, resulting in increased levels 8-hydroxydeoxyguanosine in the DNA [83]. Complicating matters, 4-HNE is more stable than other oxidative species, with a physiological half-life on the order of 2 min [84]. This allows it to diffuse and damage structures at greater distance from the site of production, relative to conventional reactive oxygen species (ROS) which are typically the species of concern when oxidative stress is considered. These ROS species, often thanks to enzymatic reduction, have half-lives on the order of milliseconds, microseconds, or nanoseconds [85,86]. Since neurons are generally non-dividing cells, DNA damage is less likely to be as immediately lethal as it is to dividing cells; however even in neurons accumulated DNA damage will begin to impair neuronal functioning over time [75]. Thus, it is plausible that Rlip depletion in neurons would result in 4-HNE accumulation which not only increases DNA damage, but also prevents its effective repair.

Overall, it is likely that Rlip plays a key role in the management of oxidative stress in neurons. Thus, loss of Rlip may elevate oxidative stress via both the increased release of ROS and decreased detoxification of oxidative fatty acid metabolites like 4-HNE. The detrimental effects of 4-HNE in neurons, combined with the established role of Rlip in the cellular efflux of 4-HNE, are alone sufficient to argue the need for further study of Rlip in the context of neurodegenerative disorders.

## 8. Neurotransmission: Rlip in Endocytosis, Exocytosis and Receptor Regulation

We recently examined the synaptic proteins synaptophysin and PSD95 and found significant reductions of both proteins in the brains of Rlip^+/−^ mice relative to WT mice, likely indicating poor synaptic health [14]. This result should not be surprising, because Rlip is a key mediator of CDE, which is used by neurons to balance presynaptic membrane re-uptake following neurotransmitter exocytosis and to regulate postsynaptic receptor densities, respectively.

**Figure 3:**
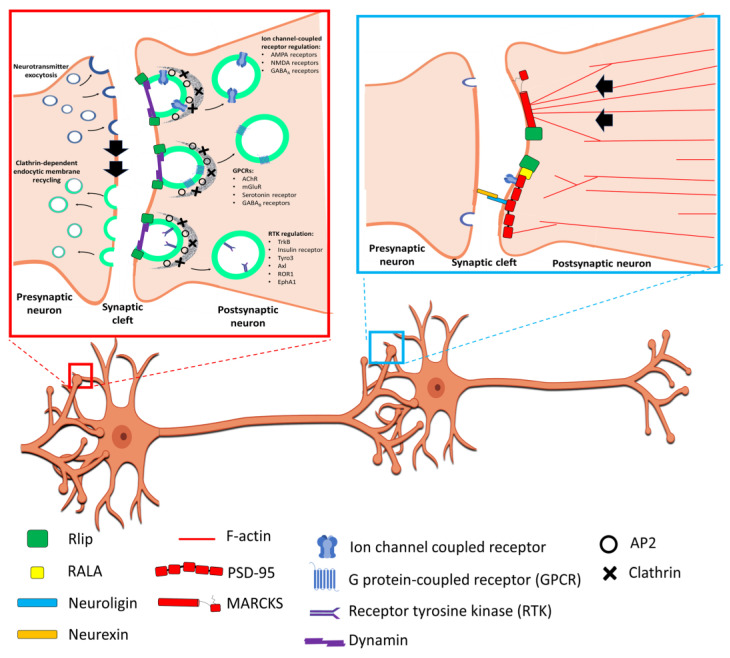
Model of Rlip in receptor regulation, membrane re-uptake, and neurite outgrowth. The left inset (**red box**) depicts roles of the Rlip and clathrin-dependent endocytosis (CDE) at a synapse. Rlip is required for CDE. Presynaptically, membrane deposited at the axon terminal following neurotransmitter exocytosis must be taken up. CDE is one method that neurons use to achieve this. Postsynaptically, the cell surface expression of many receptors is regulated by CDE. This can affect ion channel-coupled receptors, G protein-coupled receptors (GPCR), and receptor tyrosine kinases (RTK). The right inset (**blue box**) depicts the interaction of Rlip in the formation and maintenance of synapses. MARCKS plays a role in actin coordination and neurite outgrowth, while PSD-95 carries out several functions including extracellular and intracellular synaptic anchoring and receptor regulation.

Neurotransmitter exocytosis in response to an action potential is perhaps *the* hallmark characteristic of a neuron. Rlip has been reported to have roles in several cellular mechanisms which require membrane fusion events, including CDE and exocytosis [1]. As a consequence of its participation in CDE, Rlip also plays a role in the regulation of membrane receptors via endocytic internalization. Endocytosis and receptor regulation are critical to neurotransmission. In addition to the regulation of receptor densities on post-synaptic membranes, cells must balance the quantity of vesicle membrane fused to the plasma membrane at presynaptic axon terminals during neurotransmitter exocytosis with membrane quantities taken up by endocytosis [87,88]. The effects of Rlip on membrane re-uptake and receptor regulation are modeled in Figure 3 (left-hand inset).

Fortunately for Rlip KO mice, neurons possess redundant mechanisms for removing excess membrane from axon terminals. These include CDE, activity-dependent bulk endocytosis, and ultrafast endocytosis [89]. It is, therefore, unlikely that diminished CDE resulting from Rlip knockout would result in ever-ballooning axon terminals (a supposition that is supported by the very viability Rlip KO mice); however, it is still probable that changes in Rlip expression or functionality could affect the regulation of both the presynaptic and postsynaptic neurotransmission machinery, thus altering the fine tuning of neuronal signaling. Indeed, there is some evidence in the literature to indicate that this is the case. Bae et al. (2013) reported that Rlip KO decreased seizure threshold and made mice more sensitive to the convulsant drug pentylenetetrazol [90]. The authors attributed this not to an increase in neuronal excitability, but to a loss of inhibitory GABAergic interneurons in the hippocampus. Loss of these interneurons has also been associated with Alzheimer’s disease pathology [91]. Beyond the loss of the GABAergic interneurons, GABA receptors are regulated by clathrin-dependent endocytosis [92,93].

In a parallel but related line of research, Rlip, in conjunction with Ral, has also been found to regulate endocytosis of the Activin type II receptors [94]. Interestingly, activin, a regulatory protein in the TGFβ family, not only regulates brain circuits under normal physiological conditions, but is also neuroprotective against acute and chronic brain damage [95]. Impaired activin signaling has been associated with Parkinson’s disease and Alzheimer’s disease as well as anxiety disorders, depression, and drug dependence [95]. Recent evidence indicates that activin promotes neuroprotection by regulating the balance of synaptic and extrasynaptic NMDA receptors [95,96]. Providing another connection to Rlip, NMDA receptors are also regulated by CDE and are thought to be the primary culprit in glutamate excitotoxicity, a process which is associated with brain damage in Alzheimer’s disease and following ischemic brain injury [97,98]. Circling back to GABA, GABAergic signaling is thought to help keep such excitotoxity in check, as suggested by the results described in the previous paragraph [90,99]. Thus, several lines of evidence, both through direct studies involving Rlip, and through studies on CDE-dependent processes, suggest that disruption of Rlip may have direct effects at both the presynaptic and postsynaptic sides of the synaptic cleft, in addition to downstream effects arising from elevated oxidative damage.

## 9. Learning and Memory: Rlip and the Cytoskeleton in Axonal and Dendritic Remodeling

There are multiple lines of evidence that suggest that Rlip is important for cytoskeletal functions which mediate the growth of axons and dendrites. In the cancer research literature, Ras is one of the most infamous oncogenes, driving invasion and metastasis. These processes rely on mechanical forces derived from actin-dependent cytoskeletal remodeling to extend invadopodia into the surrounding tissue [100]. RalA and RalB are Ras effectors [101] which also regulate Rlip [8,9]. Rlip, as might be expected for a downstream Ras effector, has been found to be important for metastasis [102]. The GTPase activating domain of Rlip is important for regulating Rac, Rho, and Cdc42 [103,104], downstream G-protein effectors of Rlip which are also master regulators of cytoskeletal remodeling and coordinate the movement of the cytoskeleton with consequent effects on organelles, vesicles, and the plasma membrane structures such as invadopodia [26,105,106]. Similar cytoskeletal processes are instrumental to neurite outgrowth, which depends on the generation of mechanical forces by the opposing migration of actin and microtubule cytoskeletal components [107]. New axo-dendritic connections formed in this way are instrumental in learning and memory in the mature brain [108]. Neurite outgrowth has been likened to white bloods cells of the immune system, which are experts at extravasation and tissue invasion, as resembling a ‘leukocyte on a leash’ [107]. Others have noted the similarity between the invasive podosomes of immune cells and the invadopodia of cancer cells, which are also expert at extravasation and invasion [109,110], indicating the functional commonality underlying these diverse cellular behaviors.

Studies conducted using neurons have found that Rlip associates with MARCKS, a protein that regulates neurite outgrowth via CDC42 network interactions [111]. Consistently, it has also been found that the Ras and Rho GTPases are important for axon and dendrite growth and guidance [112]. Additionally, Han et al. (2009) found that that Rlip is recruited to dendritic spines by activated RalA, where it interacts with the synaptic protein PSD-95 [23]. This interaction of Rlip with PSD-95 increased by NMDA receptor activation, and Rlip was also found to be required for NMDA-induced AMPA receptor endocytosis which results in long-term depression, an important neuronal process for learning and memory [23]. Dendritic spines serve as docking points for presynaptic axons, and healthy turnover of dendritic spines depends on cytoskeletal proteins [113].

The loss of dendritic spines is a characteristic of the brains of AD patients, indicative of synaptic failure and a loss of robust neural networking [113]. A role for Rlip in synaptic health and neurite outgrowth is depicted in Figure 3 (right hand inset). As with CDE at the presynaptic and postsynaptic terminals, a combination of direct evidence from studies of Rlip in neurons and inferences taken from the findings of studies on non-neuronal cells points toward a reasonable likelihood that disruption of Rlip functionality may impact a neuron’s capacity to fine-tune its axo-dendritic connections, contributing to impoverished neural networking, neurodegeneration, and cognitive abnormalities. We have also seen that Rlip is a component of dendritic spines, which are points of axo-dendritic contact. Based on this, we hypothesize that loss of Rlip would affect axo-dendritic networking, possibly contributing to cognitive disorders.

## 10. Rlip Gene Polymorphisms and Mechanistic Links

It is currently unclear whether Rlip polymorphisms commonly exist in human populations. Leschziner et al. (2007) genotyped 503 UK epilepsy patients for 23 common Rlip polymorphisms. Among these patients they found 16 polymorphisms, of which three had a frequency of less than 1%. Across the 13 remaining polymorphisms, they found no significant associations with epileptic drug response [114]. This study included a mix of intronic and exonic polymorphisms and it is unclear how many, if any, resulted in coding changes affecting Rlip protein. By contrast, Sutiman et al. (2016) genotyped 100 people of Chinese descent, 100 of Malay descent, and 100 of Indian descent who were living in Singapore. Among the 300 subjects, the authors found no polymorphisms in exons or at exon-intron boundaries [115]. Such a result would be consistent with the high degree of conservation of the *RALBP1* gene between species, suggesting that Rlip protein has been highly optimized by evolution to carry out important functions, and loss of these functions by mutation results in diminished reproductive fitness and the loss of such polymorphisms from the gene pool. This result does not rule out the existence of rare variants or the possibility of effects from noncoding regions, perhaps in upstream promoters. In any case, together, these two studies suggest that polymorphisms which affect the function of Rlip are rare. This is consistent with research on breast cancer which also found that mutations or deletions of Rlip are exceedingly rare [116]. If changes in Rlip contribute to neurodegenerative or cognitive disorders in human patients it would likely be due to epigenetic changes in the regulation of Rlip (histone modifications or promoter methylation), altered expression of Rlip-regulating transcription factors or micro-RNAs, or possibly mutations in one of Rlip’s many interaction partners, rather than coding mutations in the *RALBP1* gene itself.

## 11. Conclusions

In summary, while there are few studies directly focused on the role of Rlip in cognitive or neurodegenerative disorders, Rlip does participate in many functions which are required by neurons. It is, therefore, reasonable to hypothesize that loss of Rlip in human patients would affect cognition and perhaps the progression of diseases like AD. Evidence indicates that mitochondrial dysfunction, oxidative DNA damage, synaptic dysfunction, and impaired neurite outgrowth, which have all been implicated in AD and other neurodegenerative disorders, are likely influenced to some degree by Rlip. The CBP/p300 transcriptional co-activators are a conserved mechanism for responding to mitochondrial stress, and the loss of function of CBP/p300 is associated with familial AD and neuronal apoptosis [117,118,119,120,121]. It has also been found that p300 regulates the expression of Rlip [122]. Thus, it is highly probable that there are subsets of AD patients that do lack proper Rlip expression. Interestingly, despite the many important cellular functions which involve Rlip, Rlip KO mice are viable. It is possible that the phenotypic effects are diminished to some degree by functional redundancies, as is the case for 4-HNE efflux or membrane endocytosis at axon terminals, as described above. It is also possible that for many of the reported functions of Rlip, Rlip serves more of a scaffolding function, enhancing the probability that other proteins will interact to carry out a given function, but not entirely necessary for the interaction to occur. While much is known about which cellular processes are associated with Rlip, little is known about how the protein machinery of Rlip carries out these functions. The 4-HNE transport function of Rlip has been demonstrated using purified Rlip protein, but for the other functions, a scaffolding role is likely more applicable. Future studies to investigate the potential neuroprotective effects of Rlip are warranted.
